# A Novel Dry-Stabilized Whole Blood Microsampling and Protein Extraction Method for Testing of SARS-CoV-2 Antibody Titers

**DOI:** 10.3390/vaccines10101760

**Published:** 2022-10-20

**Authors:** Patrick McCarthy, Joseph A. Pathakamuri, Daniel Kuebler, Jocelyn Neves, Madison Krohn, Michael Rohall, Isaac Archibeque, Heidi Giese, Martina Werner, Eugenio Daviso, Ulrich Thomann

**Affiliations:** 1 Covaris, LLC., Woburn, MA 01801, USA; 2 Department of Biology, Franciscan University, Steubenville, OH 43952, USA

**Keywords:** COVID-19, SARS-CoV-2, anti-SARS-CoV-2 IgG, Adaptive Focused Acoustics (AFA), ELISA, fingerstick blood, stabilized desiccated whole blood, decentralized blood collection, truCOLLECT, phlebotomy

## Abstract

The COVID-19 pandemic has revealed a crucial need for rapid, straightforward collection and testing of biological samples. Serological antibody assays can analyze patient blood samples to confirm immune response following mRNA vaccine administration or to verify past exposure to the SARS-CoV-2 virus. While blood tests provide vital information for clinical analysis and epidemiology, sample collection is not trivial; this process requires a visit to the doctor’s office, a professionally trained phlebotomist to draw several milliliters of blood, processing to yield plasma or serum, and necessitates appropriate cold chain storage to preserve the specimen. A novel whole blood collection kit (truCOLLECT) allows for a lancet-based, decentralized capillary blood collection of metered low volumes and eliminates the need for refrigerated transport and storage through the process of active desiccation. Anti-SARS-CoV-2 spike (total and neutralizing) and nucleocapsid protein antibody titers in plasma samples obtained via venipuncture were compared to antibodies extracted from desiccated whole blood using Adaptive Focused Acoustics (AFA). Paired plasma versus desiccated blood extracts yields Pearson correlation coefficients of 0.98; 95% CI [0.96, 0.99] for anti-SARS-CoV-2 spike protein antibodies, 0.97; 95% CI [0.95, 0.99] for neutralizing antibodies, and 0.97; 95% CI [0.94, 0.99] for anti-SARS-CoV-2 nucleocapsid protein antibodies. These data suggest that serology testing using desiccated and stabilized whole blood samples can be a convenient and cost-effective alternative to phlebotomy.

## 1. Introduction

The global Coronavirus Disease 2019 (COVID-19) pandemic has had a profound effect on industry, healthcare, and the individual due to its severe impact on human health [1,2,3]. Symptoms and severity of COVID-19 can be mitigated through the administration of either an mRNA or viral vector vaccine which confers resistance against SARS-CoV-2 by generating an immune response against the viral spike (S) glycoprotein [4]. In contrast, SARS-CoV-2 infection will trigger the generation of antibodies targeting not only S-protein, but also other viral proteins including the nucleocapsid (N) core [5]. By measuring antibody titers against these viral proteins, immunity conferred by vaccine or natural infection can be determined in both the clinical space as well as in serological studies.

Contrary to PCR tests which are used to identify ongoing infection with SARS-CoV-2, serology tests are employed to provide information about vaccination efficiency, previous exposure, and severity of disease [6]. The Centers for Disease Control and Prevention (CDC) are utilizing these antibody tests as a means of surveillance to investigate how the virus is spreading throughout populations in the United States, and to learn more about the epidemiology of COVID-19 [7]. Serology testing has been used successfully to diagnose or determine immunity to other diseases including rabies [8], hepatitis [9], and West Nile Virus [10]. They are also used to measure antibody titers for the purpose of monitoring ongoing disease states [11]. Regardless of the assay, sample collection for serological tests requires a professionally trained phlebotomist to draw several milliliters of blood using highly invasive methods, rapid on-site processing to yield plasma or serum, and necessitates cold chain storage to preserve unstable biomarkers [12]. Phlebotomy precludes self-collection and conventional transport/delivery by mail, results in sample waste due to overcollection of blood, and requires processing steps such as plasma separation upon arrival at the laboratory.

To prepare for future surveillance of outbreaks through the interrogation of large populations for past and present infections, there is growing interest in decentralized and convenient sample collection for serology testing [13]. Remote microsampling is an attractive strategy due to its ease of use and significantly reduced blood collection volumes (<100 µL). Current microsampling techniques such as dried blood spot (DBS) cards and swab-based blood collection can suffer from several challenges including difficult sample extraction and high variability between replicates due to unmetered specimen volumes and heterogeneity of collected blood [14,15]. Microsampling of standardized volumes is crucial for serological analysis [16]. Several commercial microsampling devices are available which rely on DBS-based or unstabilized liquid storage, and most are not designed for the extraction of large biomolecules [17]. Erstwhile attempts to iterate on current microsampling methods have resulted in poor quantitative correlations with plasma, indeterminate results, or the need for laborious, expensive, and time-consuming processing steps [18,19,20,21]. The truCOLLECT Whole Blood Collection Kit used herein addresses these challenges by enabling decentralized 50 µL whole blood collection, eliminating the need for cold chain shipping and storage, and by circumventing the processing steps required to generate plasma or serum.

**Figure 1:**
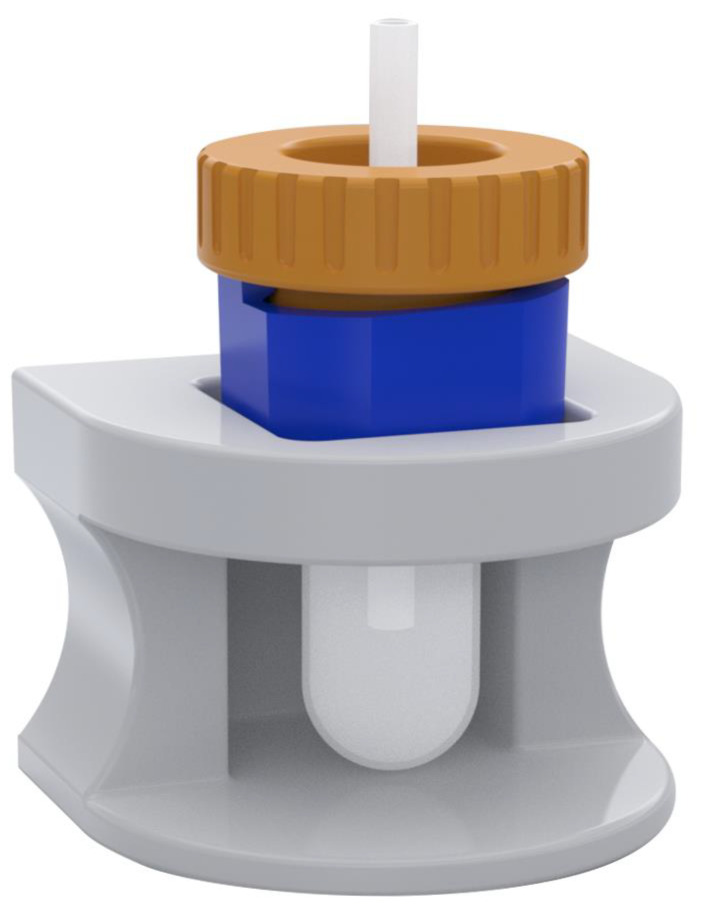
truCOLLECT Collection System. A digital drawing of the truCOLLECT collection tube and ergonomic tube holder. A protruding glass capillary allows for sample collection from a lanced finger into the tube containing an immobilization reagent.

Capillary blood is collected from a lanced finger via a K_2_EDTA-coated capillary into a collection tube containing a non-toxic immobilization reagent before undergoing active desiccation to preserve the sample for extended periods of time at ambient temperature (Figure 1). The immobilization matrix in the truCOLLECT sampling device is specifically designed to allow extraction of proteins or nucleic acids from dry-stabilized whole blood. After attaching a desiccation module, the immobilized blood sample can be shipped at ambient temperature while it is undergoing complete desiccation and hence stabilization. Extraction of biomolecules including proteins from the desiccated blood sample is then performed on demand by adding an appropriate buffer and by subjecting the sample to AFA. This process actively hydrates and homogenizes the dried blood cake and extracts proteins in less than 5 minutes which can then be processed for downstream analysis such as ELISA.

In this study, titers of antibodies against SARS-CoV-2 S- and N-protein were measured in 32 individuals, and the percentage of neutralizing S-protein antibodies determined. Blood samples were drawn by both venipuncture (followed by plasma preparation) and truCOLLECT Whole Blood (followed by on-demand plasma protein extraction via AFA), and titers were compared directly between methods.

## 2. Materials and Methods

### 2.1. Adaptive Focused Acoustics

**Figure 2:**
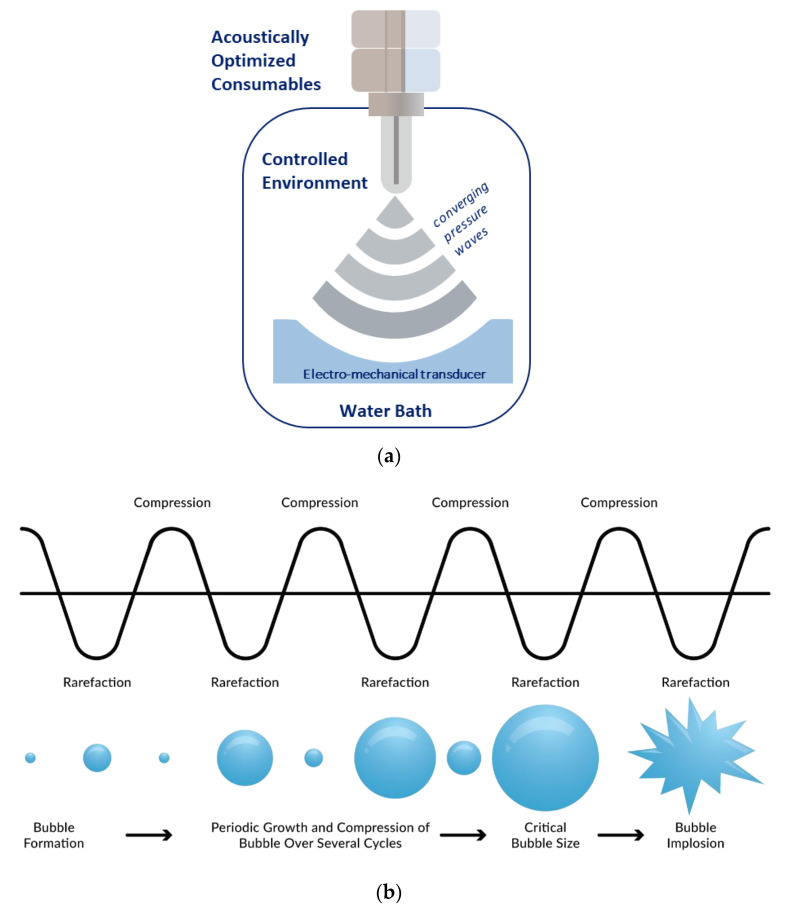
Adaptive Focused Acoustics Fundamentals. Transfer of acoustic energy from a focused-ultrasonicator transducer through a water bath and into a sample (**a**). Generation, oscillation, and collapse of microscopic bubbles in aqueous samples (**b**).

AFA is the driving force behind sample extraction from blood collected with the truCOLLECT device. AFA is a highly tunable method of ultrasonication whereby ultra-high frequency electronics and transducers produce and focus acoustic waves to effect change in a sample (Figure 2a). Energy fluctuations are manipulated to cause the formation of microscopic bubbles in dissolved gasses that grow, oscillate, and collapse (Figure 2b). This process results in localized pressure changes that can be adapted to gently mix samples, disrupt biomolecule complexes, or even fragment macro molecules. AFA treatment is completely non-contact which reduces the risk of contamination and offers a high degree of thermal control to maintain sample stability. Focused-ultrasonicator instruments can be formatted to process specimens in single tube or SBL plate formats (e.g., 24, 48, and 96 tubes), allowing for complete automation of workflows.

### 2.2. Blood Donor Demographics and Ethics

A cohort of 32 volunteers (Table 1) was recruited for this study, aged 20 to 67 years. The group consisted of 17 (53.1%) unvaccinated individuals and 15 (46.9%) people who received at least one dose of a SARS-CoV-2 mRNA vaccine. Positive tests previously verified that 17 (53.1%) participants had contracted and have since recovered from COVID-19. Of the unvaccinated volunteers, 9 (28.1%) have never tested positive based on personal communication. This study was approved by the Institutional Review Board (IRB) at Franciscan University of Steubenville (IRB #2022-05). All research was performed in accordance with relevant guidelines and regulations. Informed written consent was obtained from all participants.

### 2.3. Venipuncture Blood Sample Collection and Processing

Venipuncture blood samples were collected by phlebotomists with K-Shield Advantage Winged Blood Collection Sets (Kawasumi, Tokyo, Japan) into EDTA Vacutainer Tubes (Becton Dickinson, Franklin Lakes, NJ, USA). A total volume of 6 mL was collected from each donor. Samples were processed by centrifugation of whole blood for 10 min at 2400 rpm at 4 °C. Plasma was collected from the supernatant and stored at −80 °C prior to analysis. Sample collection was between April 2022 and June 2022.

### 2.4. Capillary Blood Sample Collection

The truCOLLECT Whole Blood Collection Kit (Covaris) was utilized to collect an exactly metered volume of capillary (fingerstick) blood via a 50 µL K_2_EDTA-coated capillary. The blood storage vessel also serves as the extraction device, thereby minimizing sample loss and avoiding tracking issues. This kit is registered as Class I device and contains an instruction manual (IFU), collection vessels, desiccant caps, gauze pads, alcohol wipes, disposable lancets, a pump dispenser, and adhesive bandages. Briefly, a contact-activated lancet was used to puncture the fingertip. After wiping the first drop of blood with gauze, blood was then applied directly to the capillary protruding from the collection device. After the capillary was filled completely, blood was dispensed into the collection tube, and the desiccant cap was used to close the device which was stored for at least 24 hours and up to 2 weeks at ambient temperature. All truCOLLECT samples were stored at ambient temperature until processing.

Extended stability (quantity and quality) has been shown for genomic DNA extracted from truCOLLECT samples stored at room temperature for 26 weeks; the time course is continuing. A parallel stability study for proteins such as IgG was started only recently. No significant difference in total protein yield was determined when extracting after 1 month of storage. However, such studies must be extended to address specific protein/plasma biomarker stability, as results may be variable depending on intrinsic characteristics of specific proteins. A study was recently started which focuses on an inflammatory biomarker panel with results pending.

**Figure 3:**
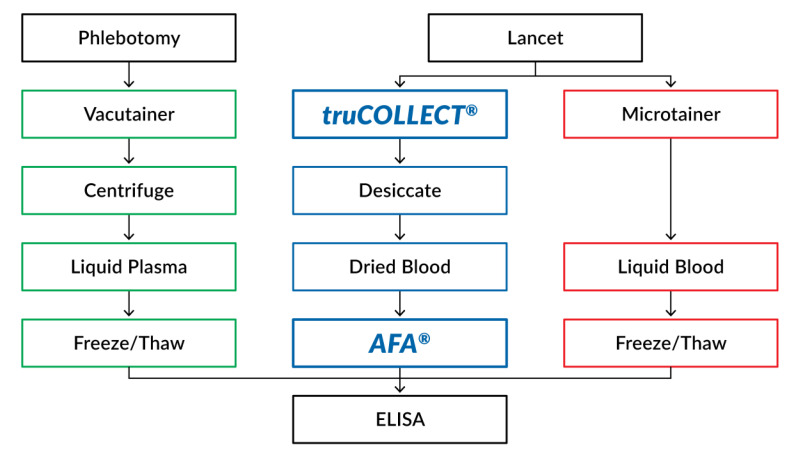
Experimental Workflow Diagram. A flowchart summarizing the three workflows tested for the processing of collected samples.

As an additional comparator, capillary blood samples (from fingerstick) were also collected using Microtainer MAP Microtubes containing K_2_EDTA (Becton Dickinson). These whole blood samples were briefly stored at room temperature prior to analysis. A workflow diagram for all sample types is presented in Figure 3.

### 2.5. truCOLLECT Sample Processing

Desiccated truCOLLECT whole blood samples were processed according to manufacturer protocols. After removal of the desiccant-containing cap, 350 µL of ELISA buffer (1X PBS, 0.05% Triton X-100) was added to the desiccated blood cake and directly subjected to AFA treatment in an M220 Focused-ultrasonicator (Covaris, Woburn, MA, USA). The extraction process utilized an average incident power of 52.5 J with a repetitive pulsing configuration. A method consisting of 40 pulsing iterations resulted in a total time of less than 5 minutes per sample. After AFA treatment, samples were centrifuged for 5 minutes at 5000× *g*, and 300 µL of the supernatant was transferred to clean microcentrifuge tubes. All samples not used directly for ELISA were stored at −80 C prior to analysis. Frozen samples were thawed to room temperature and vortexed before running serological assays.

### 2.6. Serology

Serological analysis was performed by ELISA assays targeting human IgG antibodies against SARS-CoV-2 S-protein and SARS-CoV-2 N-protein (Abcam, ab275300 and ab274339, respectively), as well as neutralizing antibodies against SARS-CoV-2 S-protein (ThermoFisher, Waltham, MA, USA, BMS2326). The ELISA kits were designed for use with serum or plasma. All samples were diluted to the degree recommended by manufacturer protocols (1:1000 for the S- and N-protein ELISA, and 1:50 for the neutralization assay), and the included controls and standards were used. The assays were run following manufacturer protocols using 100 µL controls and diluted samples, and absorbances were measured with a microplate reader (Tecan, Männedorf, Switzerland, Infinite M200pro). Replicates were run with the N protein kit to confirm the correlation served between liquid blood and truCOLLECT (data not shown). To compare results more accurately across data sets, threshold values were calculated from the average absorbances of confirmed negative donors plus 2 standard deviations (Table 1).

### 2.7. Analytical Analysis

Raw absorbance was generated by subtracting reference wavelength (570 nm) values from measurement wavelength (450 nm) values. Average absorbances were calculated from replicates for controls, standards, and samples. The COVID-19 S-Protein ELISA utilized a calibrator control to determine relative antibody concentrations, while the COVID-19 N-Protein ELISA employed a standard curve. The percentage of neutralizing antibodies was calculated from the SARS-CoV-2 Neutralizing Antibody ELISA by comparing sample absorbances with that of negative controls supplied with the kit. Plasma-derived data was plotted against both whole blood (fingerstick collected into Microtainer MAP Microtubes) and truCOLLECT Whole Blood data, and relationships were determined by calculating Pearson’s correlation coefficient with 95% confidence intervals. A direct comparison between absorbance values of different sample types was calculated by determining the ratio of paired donor samples.

## 3. Results

### 3.1. anti-SARS-CoV-2 Spike Protein Titer Analysis

**Figure 4:**
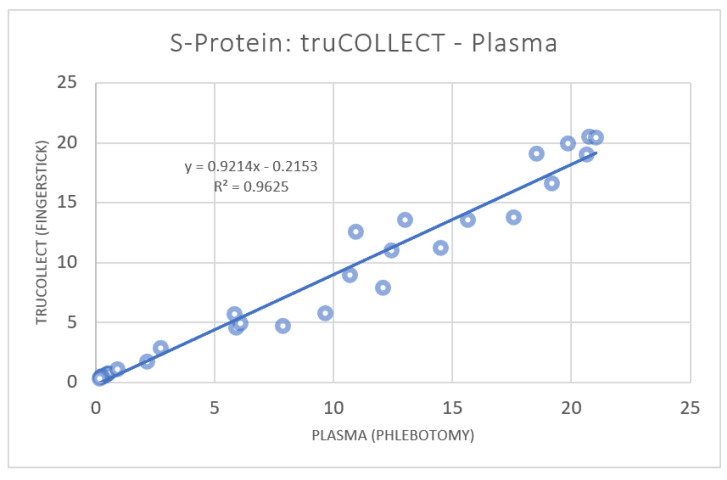
Correlation of SARS-CoV-2 S-protein antibodies in paired plasma and truCOLLECT samples. A comparison of absorbance measurements from 31 donors using the SARS-CoV-2 Spike Glycoprotein ELISA kit (Abcam). Capillary fingerstick blood collected with truCOLLECT (Y-axis), and Venipuncture derived plasma (X-axis) are examined; r 0.981; 95% CI [0.96, 0.99].

**Figure 5:**
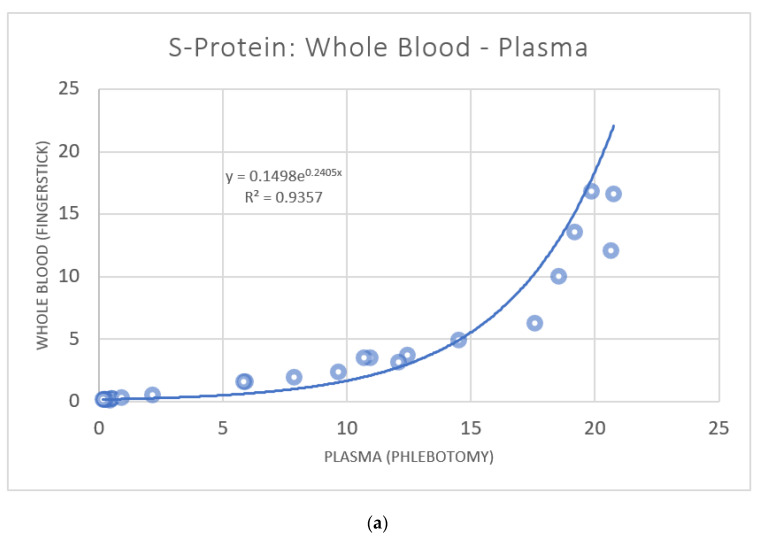

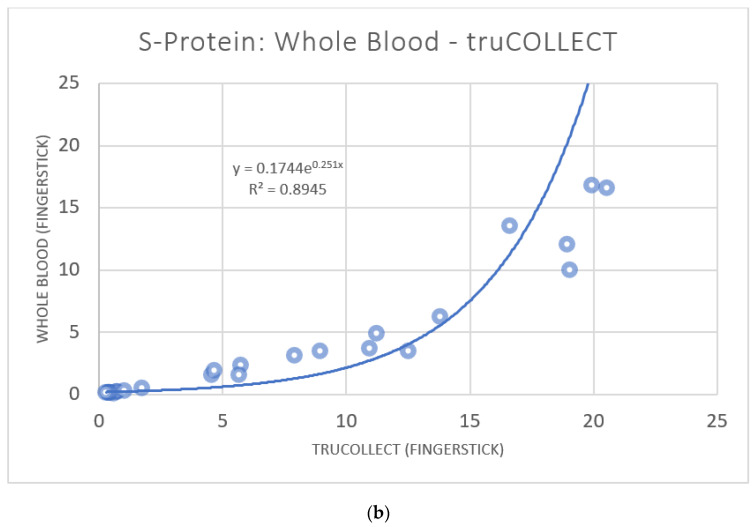
Correlation of SARS-CoV-2 S-protein antibodies in paired plasma and whole blood samples (**a**) and paired truCOLLECT extract and whole blood samples (**b**). A comparison of absorbance measurements from 31 donors using the SARS-CoV-2 Spike Glycoprotein ELISA kit (Abcam). Capillary fingerstick blood collected with Microtainer MAP Microtubes (Y-axis), and Venipuncture derived plasma (X-axis) are examined; r 0.898; 95% CI [0.78, 0.95] (**a**). Capillary fingerstick blood collected with Microtainer MAP Microtubes (Y-axis), and truCOLLECT extract (X-axis) are examined; r 0.934; 95% CI [0.86, 0.97] (**b**).

ELISA analysis of donor samples resulted in the detection of antibodies against SARS-CoV-2 S-protein in 21 (67.7%) volunteers. Of the 10 donors testing negative, one had a self-identified case of COVID-19 in Q1 2022. Paired plasma data were plotted against truCOLLECT Whole Blood extract data (Figure 4), which resulted in a Pearson correlation coefficient of 0.98 CI [0.96, 0.98] (*p*-value < 0.0001). The absorbance values obtained from plasma and truCOLLECT extract were similar, with an average ratio of 1.06 (±0.32). Paired liquid whole blood (fingerstick Microtainer) data were plotted against both plasma data (Figure 5a) and truCOLLECT extract data (Figure 5b). These comparisons resulted in a non-linear, but exponential correlation (Pearson correlation coefficient of 0.94 and 0.89, respectively, (*p*-value < 0.0001)) suggesting interference/inhibition at low titer levels in the whole blood samples as compared to plasma and truCOLLECT. In general, measured absorbances in the ELISA assay from whole blood samples were significantly lower than both plasma samples and truCOLLECT extracts from paired donors.

### 3.2. anti-SARS-CoV-2 Nucleocapsid Protein Titer Analysis

**Figure 6:**
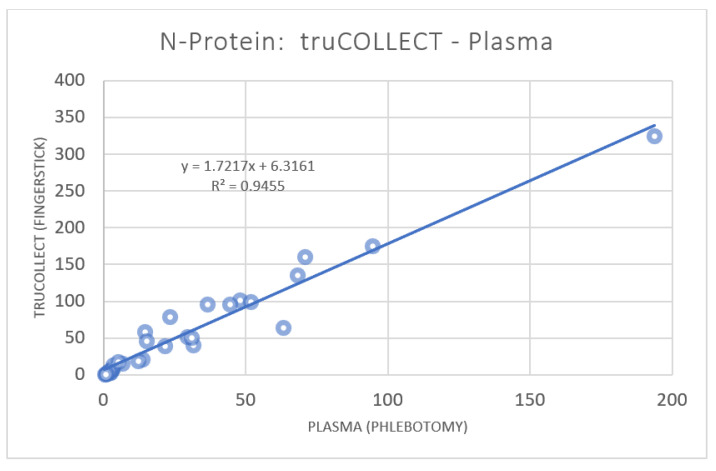
Correlation of SARS-CoV-2 N-protein antibodies in paired plasma and truCOLLECT samples. A comparison of absorbance measurements from 32 donors using the SARS-CoV-2 Nucleocapsid protein ELISA kit (Abcam). Capillary fingerstick blood collected with truCOLLECT (Y-axis), and Venipuncture derived plasma (X-axis) are examined; r 0.972; 95% CI [0.94, 0.99].

ELISA data analysis detected antibodies against SARS-CoV-2 N-protein in 19 (59%) samples using truCOLLECT Whole Blood extract or plasma (truCOLLECT and plasma NEG thresholds are 16.92 and 6.20, respectively; Table 1). Paired plasma data were plotted against truCOLLECT extract data (Figure 6) and a Pearson correlation coefficient of 0.97, 95% CI [0.94, 0.99] (*p* value < 0.001) was determined. Absorbance values for truCOLLECT-derived samples were generally higher than those obtained from plasma. Samples from donors 21 and 26 fell below the negative thresholds despite having reportedly recovered from COVID in Q1 2022 and Q3 2021, respectively. This could be due to normally waning titer levels or due to false reporting, notably for donor 21 (unvaccinated) who also has a non-detectable S-protein antibody titer. Three donors, 5, 10 and 17, show high anti-N-protein antibody titers, despite reporting no known previous COVID-19 infection.

### 3.3. SARS-CoV-2 Neutralizing Antibody Analysis

**Figure 7:**
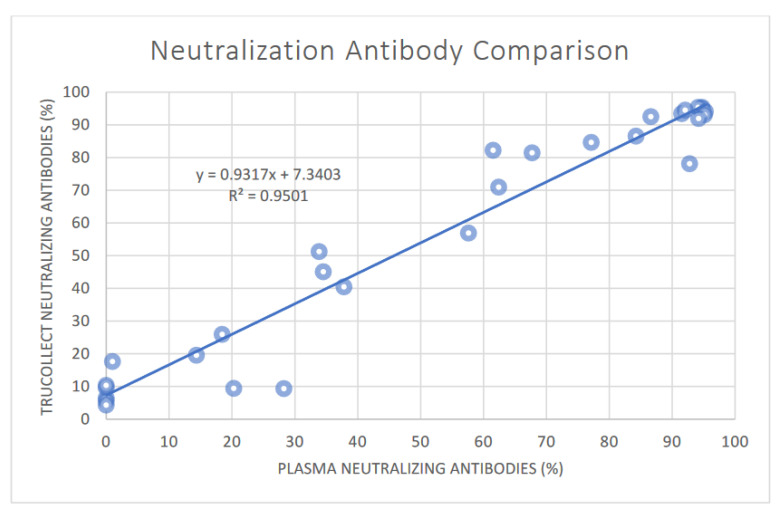
Correlation of SARS-CoV-2 neutralizing antibodies in paired plasma and truCOLLECT samples. A comparison of percent neutralization from 31 donors using the SARS-CoV-2 Neutralizing Antibody ELISA Kit (ThermoFisher). Capillary fingerstick blood collected with truCOLLECT (Y-axis), and Venipuncture derived plasma (X-axis) are compared; r 0.97; 95% CI [0.95, 0.99].

ELISA analysis of donor samples detected neutralizing antibodies against SARS-CoV-2 S-protein receptor binding domain (RBD) in 22 (68.8%) volunteers using truCOLLECT Whole Blood extract and 23 (71.9%) using plasma; one sample (donor #27) was below the negative/positive threshold (20% per test kit instructions) for truCOLLECT extract only. In the unvaccinated/COVID-19 donor group, only donor 21, who also had no detectable anti-S- or N-protein IgG titer, was negative. Interestingly, one unvaccinated donor (donor #31, Table 1) with no known COVID-19 infection had, as expected, no anti S- or N-protein IgG titer but showed a significant percentage of neutralizing antibodies. It cannot be excluded that this is a false positive due to donor specific interference with the S-protein target used in the ELISA kit. As expected, all vaccinated volunteers tested positive for neutralizing antibodies. As was the case for anti-S- and N-protein antibody titers, paired plasma versus truCOLLECT extract data (Figure 7) correlate well with an R^2^ of 0.95 (Pearson correlation coefficient = 0.97; 95% CI [0.95, 0.99] (*p* value < 0.0001)).

## 4. Discussion

The ELISA assays utilized herein were designed for use with serum or plasma. As such, the missing linear correlation between anti-S-protein IgG titers of paired plasma and whole blood samples (Figure 5) can be expected due to the potential for interference with antibody binding as well as for high background absorbance. This is corroborated by the exponential fit suggesting interference/inhibition when using whole blood as ELISA input. Conversely, the linear correlation with an R^2^ of 0.96 between paired plasma and truCOLLECT whole blood-derived titers (Figure 4) indicates that the truCOLLECT dried stabilized whole blood can be used in place of plasma samples. It should be noted that a similarly high correlation between truCOLLECT lysates and plasma was observed for other plasma proteins, including Dopamine β-hydroxylase (data not shown), suggesting that AFA-driven extraction of plasma proteins from desiccated blood using a non-denaturing buffer mainly retrieves soluble plasma proteins. Furthermore, anti-SARS-CoV-2 N-protein IgG titer measurements in truCOLLECT extracts indicate higher sensitivity in 31 (96.9%) volunteers as compared to paired plasma samples, suggesting more efficient AFA-based extraction of these specific antibodies from desiccated whole blood as compared to the plasma control. We speculate that increased sensitivity in the case of anti-N-protein IgG extracted with truCOLLECT may be due to AFA-based active mixing of the resulting extracted proteins leading to the dissociation of these specific antibodies from non-specific targets/protein complexes, thereby improving availability for binding during the ELISA; this will be a subject for future studies. However, we do not see this enhancement in the case of anti-S-protein IgG. Similar ‘enhancements’ are observable for other plasma proteins (e.g., inflammatory biomarkers) in AFA-derived truCOLLECT extracts from desiccated blood as compared to plasma controls (data not shown). The implications of these combined results are significant, suggesting a viable alternative to venipuncture.

Using truCOLLECT extracts, the humoral immunity against SARS-CoV-2 both for S and N proteins was accurately detected. When compared to self-reported patient immunity status to SARS-CoV-2, a subset of 7 (21.9%) vaccinated and unvaccinated individuals who declared no verifiable COVID-19 symptoms or known exposures to SARS-CoV-2 prior to this study tested positive for anti N-protein antibodies (vaccinated volunteers 5, 10, and 17, and unvaccinated volunteers 13 and 28; Table 1). This brings to light the growing need for rapid at-home collection of blood to elucidate real infection rates through population studies.

**Figure 8:**
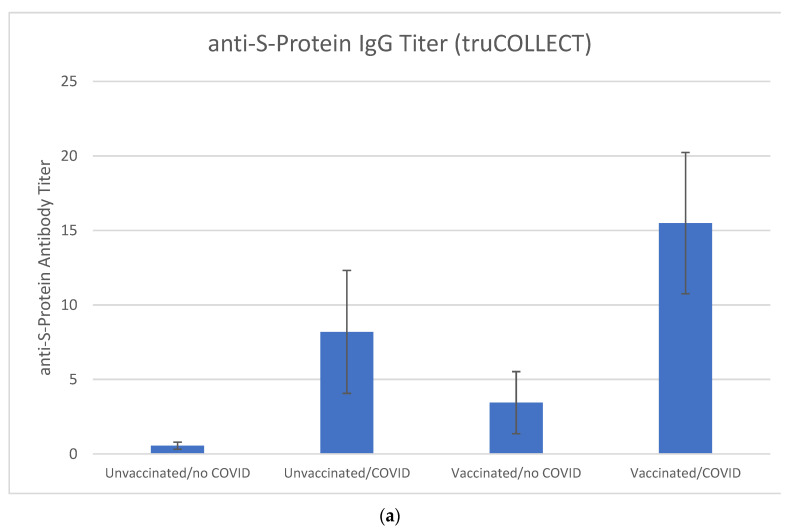

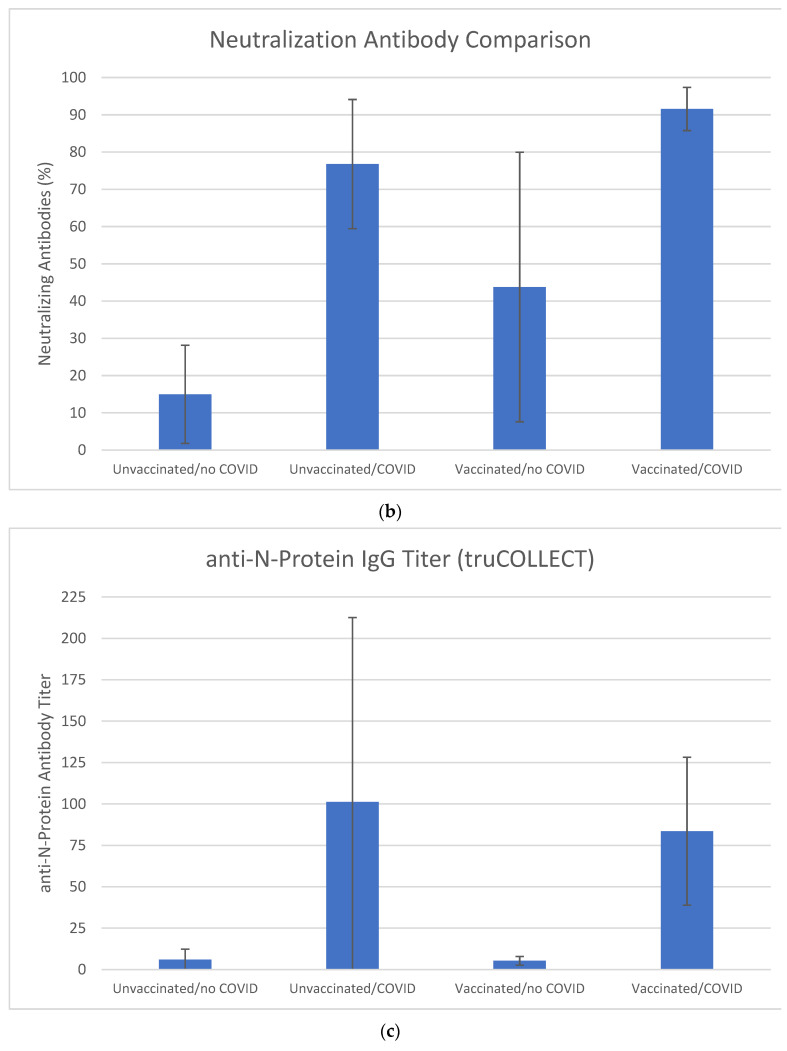
Antibody titers specific against S-protein (**a**), percent neutralization of S-protein antibodies (**b**), and antibody titers specific against N-protein (**c**) amongst different donor groups (truCOLLECT-derived extract data only): unvaccinated/no-COVID, unvaccinated/COVID, vaccinated/no-COVID and vaccinated/COVID. The weak correlation due to high variability of the N-protein IgG titers is apparent. However, both S-protein antibody titers and their respective percent neutralization levels promise to offer better potential to predict past COVID-infections.

Due to individual differences in immune response and past infection status, variations in the S-protein and N-protein specific IgG titer can be expected. There is a noticeably greater variation in anti-N-protein IgG titers as compared to anti-S-protein IgG titers, which has previously been explained as the tendency of anti-N titers to wane faster [22]. This variation in N-protein specific IgG remains significant even after regrouping certain individuals into the respective groups of unvaccinated/no-COVID, unvaccinated/COVID, vaccinated/no-COVID and vaccinated/COVID (Figure 8c). For example, unvaccinated volunteer 21, who tested positive for COVID recently, shows no detectable anti-S-protein nor anti-N-protein IgG titers as well as no neutralizing antibodies. Vaccinated volunteers 5, 10, and 17, who had no reported past COVID infection, have significant anti N-protein IgG levels and should be included in the vaccinated/COVID group. In contrast to N-protein IgG titers, after accounting for these corrections, a much clearer distinction between unvaccinated/no-COVID, unvaccinated/COVID, vaccinated/no-COVID and vaccinated/COVID groups is apparent for S-protein IgG levels and percent inactivation (Figure 8a,b). The highest titers as well as inactivation levels are observed in the vaccinated/COVID group and interestingly, the levels in the vaccinated/no-COVID and unvaccinated/COVID groups are comparable. Lastly, the linear correlation between paired plasma and truCOLLECT extract of neutralizing antibodies with R^2^ of 0.95 (Figure 7) substantiates the assertion that the antibodies extracted by AFA from desiccated blood are fully functional.

**Figure 9:**
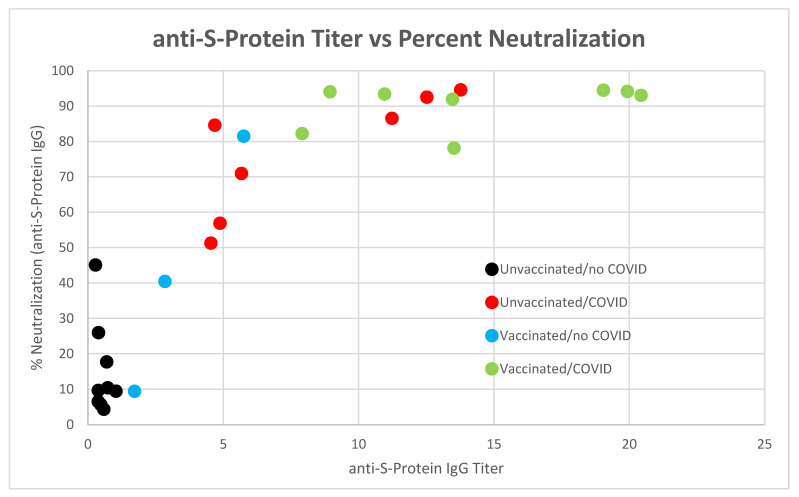
Correlation of SARS-CoV-2 neutralizing antibodies vs. anti-S-protein IgG titers in truCOLLECT and plasma samples amongst different donor groups: unvaccinated/no-COVID, unvaccinated/COVID, vaccinated/no-COVID and vaccinated/COVID.

We identified 4 clusters of data when plotting percent neutralization against anti-S-protein IgG titers as shown in Figure 9. These groups correspond to unvaccinated/no-COVID, unvaccinated/COVID, vaccinated/no-COVID and vaccinated/COVID as previously described in Figure 8. In future studies, we will increase the truCOLLECT sample size, use SARS-CoV-2 S-protein IgG titer and percent neutralization among the predictors, and select a population that is equally distributed across the identified subgroups to generate a model that will identify past COVID-19 infections as response. While SARS-CoV-2 N-protein antibody titers could be referenced for data validation, this process may circumvent the need for that measurement due to the potential volatility of anti-N titers. Consequently, utilization of S-protein antibody data alone for assessment of COVID-19 infection may be feasible. This is an intriguing prospect as underestimation of N-protein antibody data could significantly obfuscate the results of future tests or epidemiological studies, and unnecessarily complicate analyses.

## Figures and Tables

**Table 1:** Donor Information. A table summarizing demographics, vaccination and past SARS-CoV-2 infection status of volunteers, and matching ELISA data. Data are sorted by vaccination status and past COVID-19 infection information given at time of collection, ‘unvaccinated/no-COVID’, ‘unvaccinated/COVID’, ‘vaccinated/no-COVID’, and ‘vaccinated/COVID’. The units for the different ELISA data are Ratio Units (S-protein), Units/mL (N-Protein), and % (Neutralization Assay). Abbreviations: WB: Whole Blood (Fingerstick Microtainer); truCOLLECT: Whole Blood (Fingerstick truCOLLECT); Plasma: Plasma (obtained from phlebotomy-collected whole K_2_EDTA blood); M: Male; F: Female; S: Single Vaccination; D: Double Vaccination; B: Double Vaccination + Booster; ?: Unknown Vaccination Status; M/P/?: Moderna/Pfizer/Unspecified Vaccine; UV: Unvaccinated; ND: No Data; grey fields were used to calculate the NEG threshold (average negative value + 2 standard deviations).

Demographics			S antigen			Percent Neutralization		N Antigen		Vaccination		COVID
Donor ID	Age	Sex	truCOLLECT	Plasma	WB	truCOLLECT	Plasma	truCOLLECT	Plasma	State	Date	Date
1	40–49	F	0.38	0.30	0.13	6.5	0.0	2.75	1.44	UV	-	-
2	20–29	F	0.48	0.36	0.14	5.6	0.0	2.03	1.40	UV	-	-
3	50–59	F	0.59	0.45	0.02	4.3	0.0	3.44	2.49	UV	-	-
13	20–29	F	0.70	0.53	0.20	17.7	1.0	15.93	6.69	UV	-	-
14	50–59	F	0.39	0.22	0.14	9.6	0.0	1.14	0.41	UV	-	-
15	20–29	F	0.73	0.55	0.18	10.4	0.0	7.76	3.16	UV	-	-
28	20–29	F	1.04	0.91	0.26	9.4	20.3	17.33	5.18	UV	-	-
30	30–39	F	0.39	0.23	0.11	26.0	18.5	1.94	1.02	UV	-	-
31	40–49	M	0.28	0.16	0.11	45.1	34.5	1.17	0.58	UV	-	-
4	30–39	M	13.77	17.60	6.24	94.6	94.6	325.33	193.70	UV	-	Q2|2021
7	20–29	M	4.55	5.92	1.50	51.3	33.9	39.83	21.51	UV	-	Q1|2021
12	20–29	M	4.69	7.87	1.91	84.6	77.1	175.10	94.42	UV	-	Q4|2021
16	40–49	F	11.23	14.55	4.84	86.5	84.3	40.49	31.60	UV	-	Q4|2021
19	40–49	F	12.52	10.97	3.47	92.5	86.6	58.80	14.42	UV	-	Q1|2022
21	40–49	M	0.45	0.29	0.14	19.6	14.3	7.04	2.86	UV	-	Q1|2022
22	20–29	F	4.88	6.09	ND	56.9	57.6	18.77	12.07	UV	-	Q3|2021
24	20–29	F	5.67	5.82	1.55	71.0	62.4	50.43	30.95	UV	-	Q1|2021
5	60–69	F	18.97	20.65	12.01	95.0	94.7	96.22	36.62	B (M)	Q4|2021	-
9	20–29	F	5.76	9.65	2.34	81.5	67.7	7.96	3.07	S (?)	Q2|2021	-
10	20–29	F	16.62	19.22	13.51	95.4	94.8	102.25	47.84	S (?)	Q3|2021	-
17	50–59	M	20.54	20.75	16.62	95.3	94.2	96.02	44.52	B (P)	Q4|2021	-
20	20–29	F	2.85	2.77	ND	40.5	37.8	2.65	2.15	D (P)	Q2|2021	-
27	20–29	F	1.73	2.17	0.44	9.4	28.3	5.17	1.98	S (P)	Q1|2021	-
6	20–29	M	10.96	12.43	3.61	93.4	91.5	20.73	13.71	S (?)	Q1|2022	Q3|2021
8	20–29	F	19.93	19.85	16.74	94.2	94.9	64.07	63.06	D (M)	Q2|2021	Q1|2022
11	60–69	M	7.92	12.12	3.06	82.2	61.5	99.31	51.63	D (M)	Q2|2021	Q1|2022
18	30–39	M	19.05	18.56	10.03	94.5	92.1	78.66	23.18	S (?)	Q2|2021	Q4|2021
23	20–29	F	8.95	10.73	3.49	94.1	95.3	52.15	29.39	B (M)	Q4|2021	Q1|2022
25	30–39	M	20.44	21.04	ND	93.0	95.2	135.36	68.22	D (P)	Q1|2021	Q1|2022
26	20–29	M	13.53	15.70	ND	78.1	92.8	12.78	3.37	D (M)	Q3|2021	Q3|2021
29	40–49	M	13.47	13.01	ND	91.9	94.2	161.17	70.93	D (P)	Q2|2021	Q4|2021
32	60–69	F	ND	ND	ND	6.4	ND	45.66	15.21	D (P)	Q1|2021	Q1|2022
	Average Neg		0.55	0.41	0.14			5.77	2.46			
		Stdev	0.24	0.23	0.07			5.57	1.87			
Threshold Avg + 2 Stdev			**1.03**	**0.88**	**0.28**			**16.92**	**6.20**			

## Data Availability

The data presented in this study are available on request from the corresponding author. The data are not publicly available due to privacy restrictions.
